# Risk, Attributable Fraction and Attributable Number of Cause-Specific Heat-Related Emergency Hospital Admissions in Switzerland

**DOI:** 10.3389/ijph.2024.1607349

**Published:** 2024-10-07

**Authors:** Florian Schulte, Martin Röösli, Martina S. Ragettli

**Affiliations:** ^1^ Swiss Tropical and Public Health Institute (Swiss TPH), Allschwil, Switzerland; ^2^ University of Basel, Basel, Switzerland

**Keywords:** hot weather, morbidity, emergency hospital admissions, attributable risk, heat-related diseases

## Abstract

**Objectives:**

We assessed the relationship between heat and emergency hospital admissions (EHAs) in Switzerland using clinically relevant metrics.

**Methods:**

Applying distributed lag non-linear models, we investigated temperature-admission associations between May and September 1998–2019 for various disease groups, by age class and gender. We estimated the relative risk (RR) for moderate (29°C) and extreme (34°C) daily maximum temperatures relative to disease-specific optimum temperature, and calculated attributable fractions (AFs) for hot days and the following week. We also calculated the total number of heat-related EHAs.

**Results:**

We attributed 31,387 (95% confidence interval: 21,567–40,408) EHAs to above-optimal temperatures, 1.1% (0.7%–1.4%) of the total. Extreme temperatures increased the EHA risk for mental, infectious and neurological diseases. We observed particularly high AFs due to extreme heat for dehydration (85.9%, 95% CI: 82.4%–88.8%) and acute kidney injury (AKI, 56.1%, 95% CI: 45.3%–64.7%). While EHA risk generally increased with age, we also found high RRs for infectious diseases in children (0–15 years) and AKI in young adults (15–64 years).

**Conclusion:**

Hot weather increases the EHA risk in Switzerland. Therefore a comprehensive clinical and public health response is needed.

## Introduction

Exposure to high ambient temperature is an important public health hazard. Globally, about 490,000 deaths per year, or about 0.9% of the total, are attributable to heat [1]. It has been estimated that every third heat-related death between 1991 and 2018 was due to man-made climate change [2]. The most frequently documented heat-related health impacts are mortality increases attributed to cardiovascular and respiratory disease, especially among the elderly and the chronically ill [2–8].

Although heat effects on morbidity are somewhat less studied, there is a growing body of literature on this topic. Previous studies from various global regions documented increased hospital admissions [9–17], visits at emergency departments [12, 18–22] and ambulance call-outs [23–26] during periods of heat. Adverse heat effects have been found for various disease groups such as mental, respiratory, gastrointestinal or kidney diseases. The effect varies depending on local climate, age structure and the characterisation of heat exposure [4, 27–29].

In Switzerland, a small number of studies assessed the effects of hot weather on morbidity. Ragettli et al. reported an excess rate of 2.4% for emergency hospital admissions (EHAs) for non-external causes during the warm summer of 2015 [15]. For certain cardiovascular diseases such as hypertension and hear failure, however, a decrease in the risk for EHA was observed [30]. A study in the city of Bern found an increased risk for mental health hospitalisations associated with increasing temperature [14].

Most studies on heat-related morbidity do not report disease-specific attributable fractions (AFs) or absolute attributable numbers (ANs). The AFs and ANs are relevant to improve the detection of heat-related diseases in clinical settings and for public health planning. Temperature-specific AFs for relevant disease groups may assist medical teams to assess how likely heat may have caused or mimicked an emergency patient’s suspected diagnosis [31], thereby improving quality of care and informing the design of public health interventions.

This study offers a comprehensive examination of the relationship between heat and EHAs in Switzerland, employing a range of relevant metrics over a period of 22 years. We provide the attributable fraction (AF) of heat-related EHAs on a hot day and the following week, along with the total number of heat-attributable EHAs (AN) for various diagnoses and disease groups. Furthermore, we calculated the relative risk (RR) by age group and gender to identify vulnerable population groups. We also investigated whether an age group may be more susceptible for EHAs from a specific disease due to exposure to moderate or extreme heat.

## Methods

### Study Setting and Health Data

We obtained anonymized EHA data on diagnosis, age group and gender from the medical statistics of Swiss hospitals provided by the Federal Statistical Office [32]. The data set includes all emergency admissions to an emergency department of a Swiss hospital between May and September of the years 1998–2019 in the nine Swiss cantons Basel-Stadt, Basel-Land, Berne, Geneva, Lucerne, St. Gallen, Ticino, Vaud and Zurich. The area comprises the largest cities of Switzerland and the areas with the highest (urban) population density, representing 60% of the total census population in 2021. All diagnosis coding is based on the 10th revision of the International Classification of Diseases (ICD-10). The WHO version of the ICD-10 [33] was used between 1998 and 2009, and the slightly modified German version (ICD-10-GM) [34] between 2010 and 2019. The recorded ICD-10 code is aligned with the hospital discharge diagnosis, which is entered into the system by the attending physician and the hospital’s coding specialist. A high correlation between the hospital discharge diagnosis and the emergency diagnosis has been reported in high-income settings [35, 36].

We grouped data into the following disease groups (ICD-10 codes in brackets): Total EHAs (I-XXII), total non-external causes (I-XVIII excl. T67.0), certain infectious and parasitic diseases (I, A00-B99), mental and behavioural disorders (V, F00-F99), diseases of the nervous system (VI, G00-G99), diseases of the respiratory system (X, J00-J99), diseases of the digestive system (XI, K00-K93), diseases of the genitourinary system (XIV, N00-N99) and external causes (XIX-XXII & T67.0). In addition, we examined the effect of heat on the following specific renal diseases and conditions: Dehydration (E86, R39.2), urinary tract infections (N10, N13.6, N30 excl. N30.4, N39.0, N41), acute kidney Injury (N17), chronic kidney disease (N18) and kidney stones (N20-N23). Cardiovascular diseases were investigated in a previous Swiss study [30] and therefore not separately analysed. We aggregated the disease-specific EHAs among Swiss residents by canton, age group (0–14, 15–64, 65–75, 75–84 and ≥85 years), and gender (male, female).

### Temperature Data

Supplementary Table A1 shows the representative monitoring stations for each canton that was used to collect daily maximum temperature (Tmax), daily minimum temperature (Tmin) and daily mean temperature (Tmean). The temperature data was obtained from the IDAweb database, a service provided by the Swiss Federal Office of Meteorology and Climatology (MeteoSwiss) [37].

### Statistical Analysis

We explored the association between temperature and EHAs using distributed lag non-linear models (DLNMs) with conditional Poisson regression [38, 39]. DLNMs are used to describe the non-linear association between the predictor, the outcome and the lag dimension (delayed effect) by including a so-called cross-basis function in the Poisson regression model. All cantons were pooled into one data set and a stratum variable for each combination of year, month, weekday and canton was included in the model. This approach yields results equivalent to a case-control study, but can account for overdispersion and auto-correlation [39], and eliminates the need to calculate canton-specific relative risks.

### Exposure-Response Functions

We modelled the exposure dimension of the cross-basis function using a quadratic B-spline with one internal knot at the median, and boundary knots at the minimum and maximum of the warm season temperature distribution across all cantons and years. For the lag dimension of the cross-basis, we used a natural cubic spline with two internal knots equally spaced at the log scale. The maximum lag was set to 7 days, because initial analyses showed that most of the heat impact on EHAs occurs on a hot day and during the following week. We also performed an analysis with 10 days of lag for comparison. As in prior studies, the selection of the model parameters, such as knot positions, degrees of freedom and lag was based on previous research [7, 22] and preliminary analysis. To assess the robustness of our results, we performed a sensitivity analysis for total EHA risk and by diagnosis, age and gender using different temperature metrics, knots and degrees of freedom.

We run separate models by age group and gender for each disease group. Based on these models, we estimated the cumulative relative risk (RR) for EHA. The RR compares the number of EHAs on a hot day and the following week with the number of EHAs on a less hot day and the following week. We used the following temperature cut-offs: For overall heat, we compared the 99th percentile of the warm-season temperature distribution (p99) to the disease-specific reference temperature (tref). For moderate and extreme heat, we compared the 90th percentile (p90) with tref, and p99 with p90, respectively. The temperature threshold at which mortality begins to increase may vary between different disease groups and depend on age- or gender specific vulnerabilities. Therefore, we calculated separate reference temperatures for each disease-gender and disease-age group combination by identifying the minimum of the respective exposure-response function between the 25th and the 90th percentile of the warm season temperature distribution. We also calculated lag-specific RR values for each age group, gender and disease.

### EHAs Attributable to Heat

We quantified the cause-specific EHAs attributable to heat using the observed daily EHAs and the cumulative risk estimate of the exposure-response association corresponding to the observed Tmax on each day [2]. We applied this procedure to each canton-specific time series. The number of heat-related EHAs corresponds to the sum of all EHAs attributed to heat on days with Tmax above the disease-specific reference temperature over the entire observation period.

### Temperature Specific Attributable Fractions

Assuming that the whole population is directly or indirectly exposed to ambient temperature, the temperature specific RR was converted into an attributable fraction (AF) using the formula AF = (RR-1)/RR. We present the AF corresponding to the RR comparing a day with a temperature at the 99th or 90th percentile with a day with disease-specific reference temperature. In this definition, the AF represents the proportion of heat-related emergency hospital admissions on a given day with a specific temperature and the following week (lag 0–7) among all patients with the same diagnosis.

All analyses were conducted with the statistical software R (version 4.2.2), relying on the dlnm package [38].

## Results

Table 1 shows a descriptive statistic of the daily EHAs in the nine Swiss cantons during the study period 1998 to 2019 by disease, age and gender. In total, 2,884,489 EHAs were registered during the study period. Most of them (2,309,035, 80%) were due to non-external causes, especially digestive (315,076, 11%), mental (238,596, 8%) and respiratory (177,345, 6%) diseases. A considerable number of EHAs was due to external causes (575,454, 20%). Kidney stones (30,612, 1%) and urinary tract infections (31,290, 1%) made up the largest part of the examined specific kidney diseases. Most patients were female (54%) and below 65 years old (56%). Half (50%) of the EHAs were observed in the age group 15–64 years. In all age groups, external causes were the most frequent cause of EHA, followed by respiratory diseases in children 0–14 years, mental diseases in adults 15–64 years, or digestive diseases in all of the older age groups above the age of 65 years.

**TABLE 1:** Descriptive statistic of the investigated emergency hospital admissions in eight Swiss Cantons during the warm season (May-September) 1998–2019 (Risk, attributable fraction and attributable number of cause-specific heat-related emergency hospital admissions in Switzerland, Switzerland, 1998–2019).

Disease	All	0–14 years	15–64 years	65–74 years	75–84 years	≥85 years	Women	Men
Total*	2,884,489	211,143 (7.3%)	1,427,326 (49.5%)	393,317 (13.6%)	510,491 (17.7%)	342,212 (11.9%)	1,557,787 (54.0%)	1,326,702 (46.0%)
Non-external Causes*	2,309,035	140,392 (6.1%)	1,170,848 (50.7%)	326,545 (14.1%)	414,839 (18.0%)	256,411 (11.1%)	1,262,685 (54.7%)	1,046,350 (45.3%)
External Causes	575,454	70,751 (12.3%)	256,478 (44.6%)	66,772 (11.6%)	95,652 (16.6%)	85,801 (14.9%)	295,102 (51.3%)	280,352 (48.7%)
Diseases of the digestive system	315,076	18,386 (5.8%)	165,176 (52.4%)	48,276 (15.3%)	52,886 (16.8%)	30,352 (9.6%)	157,880 (50.1%)	157,196 (49.9%)
Mental and behavioural disorders	238,596	4,744 (2.0%)	186,853 (78.3%)	17,232 (7.2%)	17,927 (7.5%)	11,840 (5.0%)	123,986 (52.0%)	114,610 (48.0%)
Diseases of the respiratory system	177,345	31,512 (17.8%)	56,341 (31.8%)	28,515 (16.1%)	37,514 (21.2%)	23,463 (13.2%)	75,987 (42.8%)	101,358 (57.2%)
Diseases of the genitourinary system	148,210	8,114 (5.5%)	79,356 (53.5%)	20,168 (13.6%)	24,633 (16.6%)	15,939 (10.8%)	72,537 (48.9%)	75,673 (51.1%)
Certain infectious and parasitic diseases	136,197	20,453 (15.0%)	56,504 (41.5%)	19,501 (14.3%)	24,753 (18.2%)	14,986 (11.0%)	65,986 (48.4%)	70,211 (51.6%)
Diseases of the nervous system	84,007	4,979 (5.9%)	36,332 (43.2%)	14,411 (17.2%)	18,741 (22.3%)	9,544 (11.4%)	42,286 (50.3%)	41,721 (49.7%)
Urinary Tract Infection	31,290	2,142 (6.8%)	7,209 (23.0%)	5,255 (16.8%)	9,185 (29.4%)	7,499 (24.0%)	17,190 (54.9%)	14,100 (45.1%)
Kidney Stones	30,612	147 (0.5%)	25,292 (82.6%)	3,288 (10.7%)	1,519 (5.0%)	366 (1.2%)	8,139 (26.6%)	22,473 (73.4%)
Acute kidney injury	7,627	25 (0.3%)	1,746 (22.9%)	1,625 (21.3%)	2,616 (34.3%)	1,615 (21.2%)	3,246 (42.6%)	4,381 (57.4%)
Dehydration	5,852	557 (9.5%)	826 (14.1%)	709 (12.1%)	1,695 (29.0%)	2,065 (35.3%)	3,468 (59.3%)	2,384 (40.7%)
Chronic Kidney Disease	4,876	52 (1.1%)	1,366 (28.0%)	989 (20.3%)	1,562 (32.0%)	907 (18.6%)	2,089 (42.8%)	2,787 (57.2%)

For each disease group, the absolute number of emergency hospital admissions and its proportion in % of the disease-specific total is shown by age group and gender.

*includes cardiovascular diseases which were not the focus of this study and are studied in relation to temperature elsewhere [30].

Over all cantons, the average Tmean, Tmin and Tmax during the study period was 22.7°C, 3.2°C and 39.7°C, respectively. The populations in different cantons were exposed to similar temperatures, with canton-specific maximum Tmax ranging from 33.3°C in St. Gallen to 39.7°C in Geneva. More detailed information on the measured temperature levels in each canton is presented in Supplementary Table A1.

**FIGURE 1:**
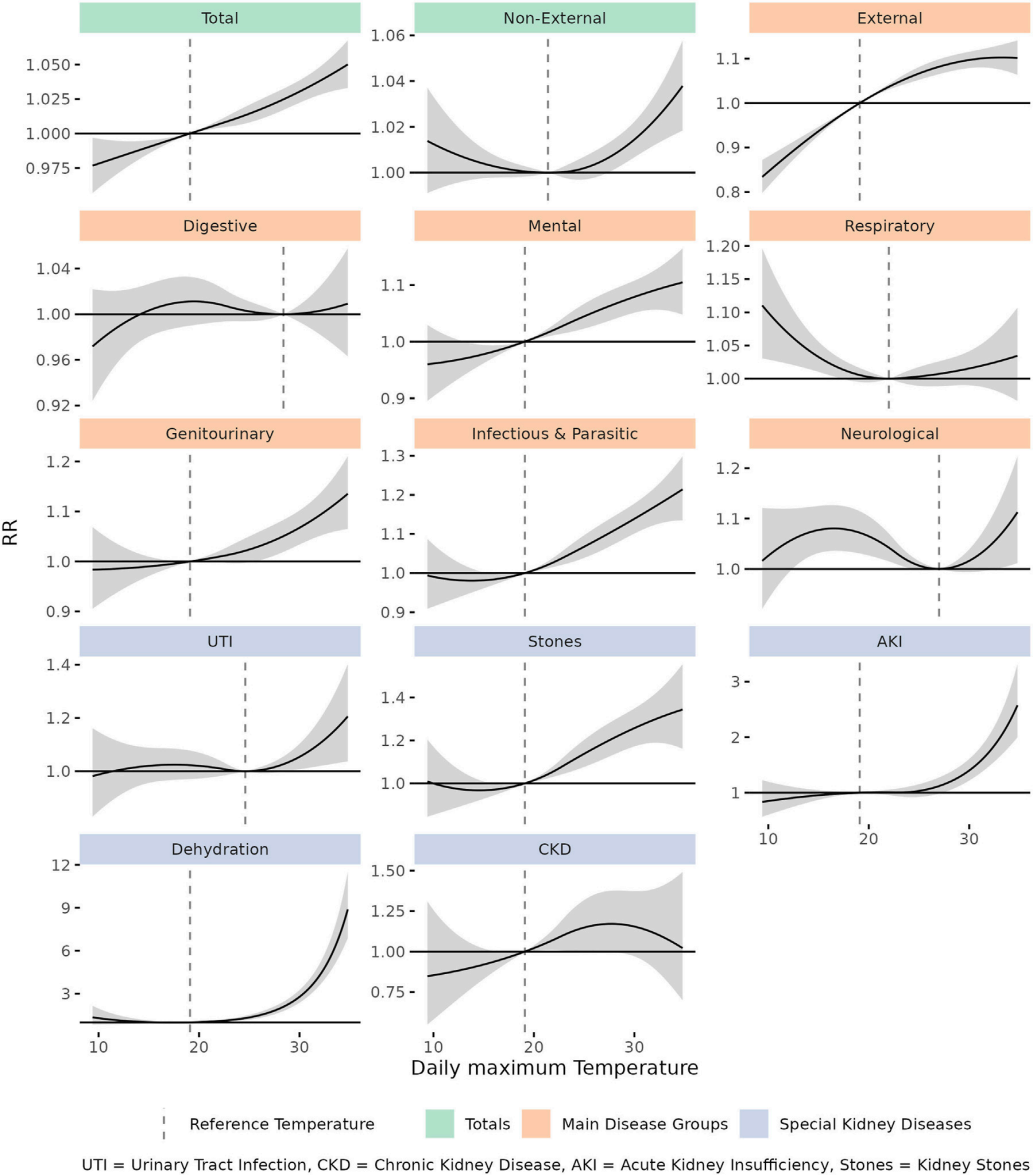
Cumulative (lag 0–7) relative risk (RR) with 95% confidence interval between daily maximum temperature and emergency hospital admissions for examined diseases during the warm season. Dashed vertical lines show the disease-specific reference temperature (Risk, attributable fraction and attributable number of cause-specific heat-related emergency hospital admissions in Switzerland, Switzerland, 1998–2019).

Figure 1 shows the exposure-response functions for all examined disease groups. On a hot day with 34°C and the following week, the risk for EHA increased by 5% (RR = 1.05, 95% CI: 1.03–1.06) compared to a day with the disease-specific reference temperature and the following week. Specifically, we observed an increase in RR with temperature for external causes, mental diseases, genitourinary diseases, infectious diseases and neurological diseases, and for all examined specific kidney diseases except for chronic kidney disease. The highest RRs were observed for dehydration (7.1, 95% CI: 5.67–8.9), acute kidney injury (2.28, 95% CI: 1.83–2.83) and kidney stones (1.33, 95% CI: 1.18–1.51).

Disease-specific RR also varied by lag. For external causes, infectious diseases and dehydration, we observed the strongest impact on lag 0. For mental diseases, AKI and kidney stones, we observed the strongest effects on lag 1–4, 1–5 and 1–3, respectively. All lag-specific RRs and attributable fractions are shown in Supplementary Figure A1.

Table 2 displays the age- and gender specific cumulative RR for all examined diseases, comparing the warm-season P99 of Tmax with the disease-specific reference temperature. In general terms, the risk increase was higher for women (6% [96% CI: 4%–8%]) than for men (3% [95% CI 1%–5%) and particularly pronounced in the very old ≥85 years (9% [95% CI: 4%–13%]). Children were most affected by heat-related EHAs classified as infectious diseases. Adults and young pensioners were often admitted for external causes during periods of heat. In the elderly, heat was associated with a considerable increase in EHA risk for dehydration and acute kidney injury. Specifically, we also observed an increase in EHAs due to urinary tract infections (UTI) in old women, and due to kidney stones in old men.

**TABLE 2:** Cumulative (lag 0–7) relative risk for emergency hospital admission with 95% confidence interval by disease group, specified by age class and gender (Risk, attributable fraction and attributable number of cause-specific heat-related emergency hospital admissions in Switzerland, Switzerland, 1998–2019).

Disease	All	0–14 years	15–64 years	65–74 years	75–84 years	≥85 years	Women	Men
Total	**1.05 (1.03–1.06)**	1.01 (0.96–1.06)	**1.05 (1.03–1.07)**	**1.05 (1.02–1.09)**	**1.04 (1–1.07)**	**1.09 (1.04–1.13)**	**1.06 (1.04–1.08)**	**1.03 (1.01–1.05)**
Non-external Causes	**1.03 (1.02–1.05)**	1.03 (0.96–1.09)	1.02 (1–1.04)	1.02 (0.98–1.07)	1.04 (1–1.08)	**1.12 (1.07–1.17)**	**1.05 (1.03–1.08)**	1.01 (0.99–1.04)
External Causes	**1.1 (1.07–1.14)**	0.98 (0.89–1.07)	**1.18 (1.13–1.23)**	**1.27 (1.17–1.38)**	1.05 (0.98–1.13)	1.02 (0.95–1.08)	**1.1 (1.05–1.14)**	**1.11 (1.06–1.16)**
Diseases of the digestive system	1.01 (0.97–1.05)	1.01 (0.86–1.19)	1.02 (0.96–1.08)	1.07 (0.97–1.19)	0.97 (0.89–1.05)	0.97 (0.85–1.1)	1.05 (0.98–1.11)	0.98 (0.93–1.03)
Mental and behavioural disorders	**1.1 (1.05–1.15)**	0.9 (0.67–1.2)	**1.09 (1.03–1.14)**	1.18 (1–1.41)	**1.2 (1.03–1.4)**	1.2 (1–1.43)	**1.08 (1.02–1.15)**	**1.12 (1.05–1.19)**
Diseases of the respiratory system	1.03 (0.97–1.09)	**0.72 (0.63–0.82)**	1.04 (0.95–1.14)	1.14 (0.99–1.32)	**1.19 (1.05–1.33)**	1.15 (0.98–1.34)	1.01 (0.92–1.1)	1.05 (0.97–1.13)
Diseases of the genitourinary system	**1.12 (1.06–1.19)**	0.95 (0.77–1.17)	**1.1 (1.01–1.18)**	0.98 (0.86–1.11)	**1.18 (1.03–1.34)**	**1.54 (1.3–1.82)**	1.08 (0.99–1.18)	**1.18 (1.09–1.27)**
Certain infectious and parasitic diseases	**1.2 (1.13–1.27)**	**1.38 (1.18–1.6)**	**1.13 (1.03–1.24)**	1.16 (1–1.34)	**1.3 (1.14–1.48)**	1.16 (0.98–1.37)	**1.26 (1.16–1.36)**	**1.15 (1.06–1.25)**
Diseases of the nervous system	**1.09 (1.01–1.18)**	**1.42 (1.01–2)**	0.97 (0.87–1.07)	1.18 (0.97–1.44)	**1.21 (1.03–1.43)**	1.11 (0.9–1.37)	**1.18 (1.05–1.32)**	1.01 (0.93–1.11)
Urinary Tract Infection	**1.17 (1.03–1.33)**	0.97 (0.61–1.54)	0.97 (0.76–1.24)	1.14 (0.83–1.55)	1.12 (0.9–1.39)	**1.62 (1.26–2.06)**	**1.36 (1.15–1.62)**	1 (0.84–1.18)
Kidney Stones	**1.33 (1.18–1.51)**	2.5 (0.4–15.76)	**1.34 (1.16–1.54)**	1.08 (0.73–1.59)	1.34 (0.79–2.27)	**6.9 (2.38–20.03)**	1.22 (0.96–1.55)	**1.37 (1.19–1.59)**
Acute kidney injury	**2.28 (1.83–2.83)**	<100 cases	**3.27 (2.08–5.13)**	1.49 (0.92–2.4)	**2.52 (1.67–3.81)**	**2.35 (1.42–3.89)**	**1.85 (1.29–2.64)**	**2.67 (2–3.57)**
Dehydration	**7.1 (5.67–8.9)**	**3.63 (1.49–8.85)**	**4.85 (2.37–9.95)**	**9.48 (5.06–17.74)**	**6.97 (4.56–10.66)**	**8.76 (6.15–12.5)**	**8.02 (6–10.71)**	**5.92 (4.14–8.45)**
Chronic Kidney Disease	1.05 (0.76–1.45)	<100 cases	1.01 (0.54–1.9)	0.97 (0.47–2.01)	0.96 (0.59–1.54)	1.13 (0.55–2.33)	1.21 (0.74–1.98)	0.94 (0.61–1.43)

Comparison between the 99th percentile of the daily maximum temperature distribution (p99, 34°C) and the reference temperature (specific for disease and demographic group). Significant values in bold.

**FIGURE 2:**
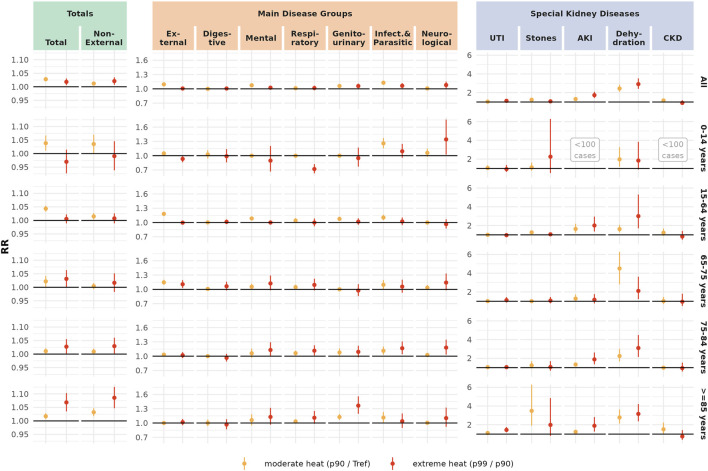
Cumulative (lag 0–7) relative risk with 95% confidence interval comparing moderate heat (p90, 29.3°C) with the reference temperature, and extreme heat (p99, 34°C) with moderate heat (Risk, attributable fraction and attributable number of cause-specific heat-related emergency hospital admissions in Switzerland, Switzerland, 1998–2019).

Effects for moderate and extreme heat by age are presented in Figure 2. Moderate heat was associated with an increase in EHA risk for total, non-external, external, mental, genitourinary and infectious diseases in various age groups. Especially the increase for external causes was mainly attributable to moderate heat, which corresponds to the flattening shape of the respective E/R-curve. Extreme heat was associated with an increasing EHA risk for total and non-external causes, especially in the oldest age group. A particularly striking additional effect of extreme heat was detectable for dehydration and acute kidney injury across age groups, consistent with the exponential shape of the respective E/R curves (Supplementary Figure A2).

Table 3 shows the estimated number of heat-related EHAs in Switzerland between 1998 and 2019 by diagnosis. According to our model, 31,387 (95% CI: 21,567–40,408) out of the total 2,884,489 EHAs were attributable to heat. Most cases were observed for external causes, mental and behavioural disorders, infectious diseases and diseases of the genitourinary system. Among the examined specific kidney diseases, kidney stones accounted for the largest number of heat-related EHAs.

**TABLE 3:** Left part: Absolute and relative heat-attributable emergency hospital admissions with 95% confidence interval by disease group. Right part: Heat attributable fractions on a hot day with 34°C and the following week with 95% confidence interval by disease group (Risk, attributable fraction and attributable number of cause-specific heat-related emergency hospital admissions in Switzerland, Switzerland, 1998–2019).

Disease	Ref. Temp	Heat-related EHA, May-September 1998–2019 on days with tmax > Ref. Temp		Fraction of heat-related emergency hospital admissions (EHA) on a hot day with 34°C and the following week after a heat event with a temperature of…	
		Heat-attributable EHA	Share on summer EHA 1998–2019	29.3°C (p90)	34°C (p99)
Total	19.1°C	**31,387 (21,567–40,408)**	**1.1% (0.7%**–**1.4%)**	**2.7% (2.0%**–**3.5%)**	**4.4% (3.1%**–**5.8%)**
Non-external Causes	21.4°C	**7,906 (1,760–13,849)**	**0.3% (0.1%**–**0.6%)**	**1.2% (0.5%**–**1.9%)**	**3.2% (1.6%**–**4.8%)**
External Causes	19.1°C	**23,962 (19,901–27,688)**	**4.2% (3.5%**–**4.8%)**	**8.6% (7.1%**–**10.1%)**	**9.3% (6.5%**–**12.0%)**
Diseases of the Digestive system	28.4°C	76 (−415–591)	0.0% (−0.1%**–**0.2%)	0.0% (−0.4%**–**0.4%)	0.7% (−3.2%**–**4.5%)
Mental and behavioural disorders	19.1°C	**7,104 (4,653–9,743)**	**3.0% (2.0%–4.1%)**	**6.9% (4.5%–9.3%)**	**9.2% (4.9%–13.2%)**
Diseases of the respiratory system	22°C	672 (−682**–**2,014)	0.4% (−0.4%**–**1.1%)	1.3% (−1.0%**–**3.5%)	3.0% (−2.8%**–**8.4%)
Diseases of the genitourinary system	19.1°C	**3,157 (1,104–5,016)**	**2.1% (0.7%–3.4%)**	**5.6% (2.7%–8.5%)**	**10.9% (5.9%–15.7%)**
Certain infectious and parasitic diseases	19.1°C	**6,374 (4,478–8,140)**	**4.7% (3.3%–6.0%)**	**11.4% (8.4%–14.2%)**	**16.7% (11.8%–21.4%)**
Diseases of the nervous system	27°C	336 (−42–697)	**0.4% (0.0%–0.8%)**	0.9% (−0.6%**–**2.5%)	**8.2% (0.6%–15.3%)**
Urinary Tract Infection	24.6°C	**387 (67–716)**	**1.2% (0.2%–2.3%)**	**3.9% (0.3%–7.4%)**	**14.7% (3.1%-24.9%)**
Kidney Stones	19.1°C	**2,548 (1,711–3,280)**	**8.3% (5.6%–10.7%)**	**19.5% (13.8%–24.8%)**	**24.9% (14.9%–33.7%)**
Acute kidney injury	19.1°C	**624 (154–1,006)**	**8.2% (2.0%–13.2%)**	**24.0% (12.8%–33.7%)**	**56.1% (45.3%–64.7%)**
Dehydration	19.1°C	**1,735 (1,453–1,973)**	**29.7% (24.8%–33.7%)**	**59.0% (52.0%–65.0%)**	**85.9% (82.4%–88.8%)**
Chronic Kidney Disease	19.1°C	**345 (11–617)**	**7.1% (0.2%–12.7%)**	14.0% (−1.6%**–**27.3%)	4.9% (−31.3%**–**31.1%)

Significant values in bold.

Alongside, Table 3 shows the disease-specific attributable fractions on a moderately (29.3°C, p90) or extremely (34°C, p99) hot day and the following week, compared with a day with disease-specific reference temperature and the following week. Following an extreme heat event (p99, 34°C), 4.4% (95% CI: 3.1%–5.8%) of all EHAs were statistically attributable to heat. Particularly high attributable fractions were observed for dehydration (85.9%, 95% CI: 82.4%–88.8%), acute kidney injury (56.1%, 95% CI: 45.3%–64.7%) and kidney stones (24.9%, 95% CI: 14.9%–33.7%) after extreme heat events, which corresponds to the marked RR increase with temperature for these diseases.

Sensitivity analyses assessing the impact of model choices on our results did not change the shape of the exposure-response functions. The exposure-response functions also looked similar for Tmin and Tmean. The results for overall (p99/tref), moderate (p90/tref) and extreme (p99/p90) heat were similar across models. Detailed plots are presented in the Supplementary Figures A3–A5.

## Discussion

Our study shows that both moderate and extreme heat is associated with an increased EHA risk in Switzerland. Overall, we estimated that 31,387 (95% CI) all-cause EHAs can be attributed to heat during the warm season 1998–2019, representing 1.1% (95% CI) of the total EHAs during the period. In absolute terms, most heat-related EHAs were diagnosed as external causes (23,962, 95% CI: 19,901–27,688), mental diseases (7,104, 95% CI: 4,653–9,743), and infectious diseases (6,374, 95% CI: 4,478–8,140). On a hot day of 34°C and the following week, 4.4% of all EHAs can be attributed to heat exposure, as well as 86% of the dehydration cases, 56% of the acute kidney injury cases and 25% of the kidney stone cases. The risk of EHA mainly increased for infectious diseases in children, for external causes in adults up to 64 years of age, and for dehydration, kidney stones and UTI in the elderly.

External causes made up the largest number of heat-attributable EHAs. The RR for EHA due to external causes increased mainly on lag 0 in the age groups 15–64 and 65–74, and was most pronounced in moderate heat. Similar to previous studies, we assume that the most likely explanation for this finding are outdoor leisure time and occupational accidents during warm summer weather, although reduced cognitive or motor function under heat stress may also contribute [15, 26, 40, 41]. Our results differ from some previous research that identified heat-related fractures mainly in the elderly [20].

For mental and behavioural disorders, there was a significant increase of the EHA risk with Tmax at lag 1–4. The effect is well described and the lag structure is well in line with similar studies from Bern, Switzerland and Toronto, Canada. The risk increase can be explained by specific heat effects, such as altered neurotransmitter levels, interference of heat with psychiatric drugs, impaired cognitive function or sleep deprivation [11, 14, 42].

Heat-related EHAs for infectious diseases increased across age groups, with children (0–14 years) most affected. Previous studies described a higher risk for gastroenteritis with heat that may account for some of these cases [12, 43]. Another study by Bobb et al. suggested that heat-related illness may mimic sepsis, which could lead to a false increase of EHAs for supposed infectious diseases [44]. There are indications that such misdiagnosis may explain some of the risk increase for infectious diseases that we observed, both in children and adults. First, heat exhaustion and heat stroke (“heat illness”) are generally overlooked. The limited available research shows low detection rates (0%–3%) especially in Europe, which means that most cases falsely receive a different diagnosis [45, 46]. Second, the symptoms of heat illness and infectious diseases overlap considerably: Hemoconcentration due to heat-induced dehydration can mimic leukocytosis, while hyperthermia, hypotension, tachycardia and impaired consciousness are characteristic for both heat illness and severe infections, particularly sepsis [47, 48]. This favours the misdiagnosis of heat-related illnesses as an infectious disease, especially sepsis. And third, we observed a similar lag pattern between dehydration, which is a direct heat effect, and infectious diseases: Both peak on day 0, with little effect on the consecutive days. This may indicate that both observations in fact describe the same underlying event. Future research may address this finding by examining patient level data, e.g., with a similar approach as used by Oberlin et al. [45].

For dehydration, acute kidney injury (AKI) and kidney stones, our results showed a particularly strong association of heat and EHAs. The affected patients were usually 85 years and older or of working age (15–64 years). The observed effect was consistent with literature [10, 12, 28, 44, 49–52] and followed an exponential (dehydration, AKI) or near-linear (kidney stones) exposure-response curve. A causal pathway from heat over sweating to dehydration and acute kidney injury has been widely described in literature and experimentally confirmed [40, 53, 54]. Similarly, the accumulation of minerals in concentrated urine can lead to kidney stones [40, 51]. With all applicable Bradford Hill criteria for causality [55] fulfilled, our results suggest that on a hot day with 34°C and in the following week, more than 4/5th of the dehydration cases, more than half of the acute kidney injury cases, and a quarter of the kidney stone cases are likely caused by heat exposure. The risk increase also affected the working-age population between 15 and 64, which could justify extending heat protection measures to this age group to reduce exposure.

We also found a heat-related increase for urinary tract infections (UTI), specifically in the very old (≥85) and in women. The risk group is unsurprising: Younger patients with (suspected) UTI would likely receive outpatient treatment and not be included in the EHA statistic, and UTI are uncommon in men. We suggest two plausible explanations for increasing EHAs due to UTI with heat. On one hand, dehydration has been described as a risk factor for UTI, especially in care-dependent individuals [56]. Several pathophysiological pathways have been proposed for this effect, although with inconsistent evidence [57]. On the other hand, missed cases of heat emergencies may also contribute, similar as for infectious diseases: Heat-induced hyperthermia may be misinterpreted as fever, while an inconclusive urine sample due to low urine output may remain the only pathological finding after diagnostic work-up for suspected infection, leading to hospitalization for UTI.

Our study provides a comprehensive assessment of the effects of heat on EHAs for different causes, subgroups and temperature ranges over an extended period of time. By quantifying the effect in the form of attributable numbers and attributable fractions, we provide relevant results for public health professionals and clinicians that may help to improve the detection of heat-related diseases. For instance, emergency medical teams can use the AF to estimate the risk that the condition of a specific emergency patient is heat-related under current meteorological conditions. We could show that heat events are associated with a substantial increase in EHAs in Switzerland. The effect already occurs in moderate heat and causes the majority of EHAs for some diseases (dehydration, acute kidney injury) on a hot day with 34°C and the following week.

Our results demonstrate the potential of primary prevention for vulnerable population groups. This could be achieved by appropriate heat protection measures, such as sufficient thermal insulation and passive cooling of buildings [58] like nursing homes, heat-resilient urban planning, and workplace safety measures such as cooling clothes and adapted working hours for outdoor workers during hot spells [59]. Further, secondary prevention should include the training of healthcare professionals in the detection and management of heat-related diseases by appropriate measures. Such measures include the inclusion of heat exhaustion and heat stroke in common differential diagnostic schemes for dizziness, elevated body temperature and altered level of consciousness. Furthermore, physical cooling should be made available as a standard practice in emergency and non-intensive care settings to improve the treatment of heat exhaustion and heat stroke. The study also identified a substantial number of EHAs due to external causes that is probably caused by behavioural changes on hot days, although the physiological effects of heat may amplify the risk. This finding should be further investigated and may require entirely different prevention measures, such as accident prevention programs.

To facilitate the implementation of prevention measures such as heat health action plans, a multi-stakeholder approach is needed, where scientific institutions, medical or nursing organizations, health governments, civil defence authorities, municipalities and occupational safety organizations collaborate with each other [40, 60]. In such a manner, feasible and acceptable solutions can be developed [61], which efficiently reduce the heat-related health burden.

However, some limitations have to be acknowledged. Inevitably, first diagnoses in hospital records may be optimized for changes in insurance reimbursement schemes, such as the introduction of diagnosis related groups (DRGs) in Switzerland in 2012 [62, 63]. However, such long-term changes do not correlate with short-term weather fluctuation and are therefore unlikely to cause bias. As in previous studies on the temperature-morbidity relationship [15], we used temperature data from only one station per canton. Thus, within-canton variability of temperature could not be accounted for. By including only the cantons with the largest urban areas, choosing representative monitoring stations and excluding the mountain area, we tried to minimize exposure misclassification. We did not account for possible confounders and effect modifiers such as air pollution or humidity. While prior research showed an independent effect of temperature on health outcomes, the additional effect of air pollution has been found to be small [4]. Heat can affect air pollution levels, in particular ozone. This implies that air pollution is on the pathway and serves as a potential mediator for heat-related health effects. Consequently, adjusting for air pollutants such as ozone would have potentially removed these indirect heat effects, leading to an underestimation of heat-related morbidity [64]. Previous research has demonstrated that heat metrics incorporating humidity, such as apparent temperature, yield comparable outcomes to tmax [7, 65, 66]. A recent large scale analysis has confirmed this for cities with a robust negative correlation between temperature and humidity [67], which is the case for Switzerland. Therefore, we selected the more accessible and comprehensible measurement to facilitate the utilisation of the study results in clinical practice.

### Conclusion

Heat accounts for a considerable amount of emergency hospital admissions (EHAs) in Switzerland. External causes, mental and behavioural disorders and infectious diseases are the most frequent heat-related diagnoses. Our findings suggest that the majority of EHAs for dehydration and acute kidney injury on a hot day with 34°C and the following week can be attributed to heat exposure, especially in the elderly and working ages. A multi-stakeholder approach is needed to develop and implement feasible and acceptable prevention measures for risk groups to reduce heat exposure and improve the detection of heat-related diseases.

## Availability of R-Code and Data

The health data used for this study are available under license from the Federal Statistical Office, Switzerland. The R code is available on request from the authors.
