# Supplementary material for: Risk, Attributable Fraction and Attributable Number of Cause-Specific Heat-Related Emergency Hospital Admissions in Switzerland
Source: Int J Public Health. 2024 Oct 7;69:1607349. doi: 10.3389/ijph.2024.1607349 (PMC11491377; doi:10.3389/ijph.2024.1607349)
Supplement: Supplementary file 1 [file DataSheet1.PDF]

Risk, attributable fraction and attributable number of cause-specific heat-related emergency hospital admissions (EHA) in Switzerland

Supplementary

Figure A1. Lag-specific Risk Ratios (RR) and attributable fractions for heat-related emergency hospital admission in Switzerland 1998-2019, comparing the 99th percentile of the daytime maximum temperature distribution (p99, 34°C) with the reference temperature. Alternative lag period (10 days) in blue.

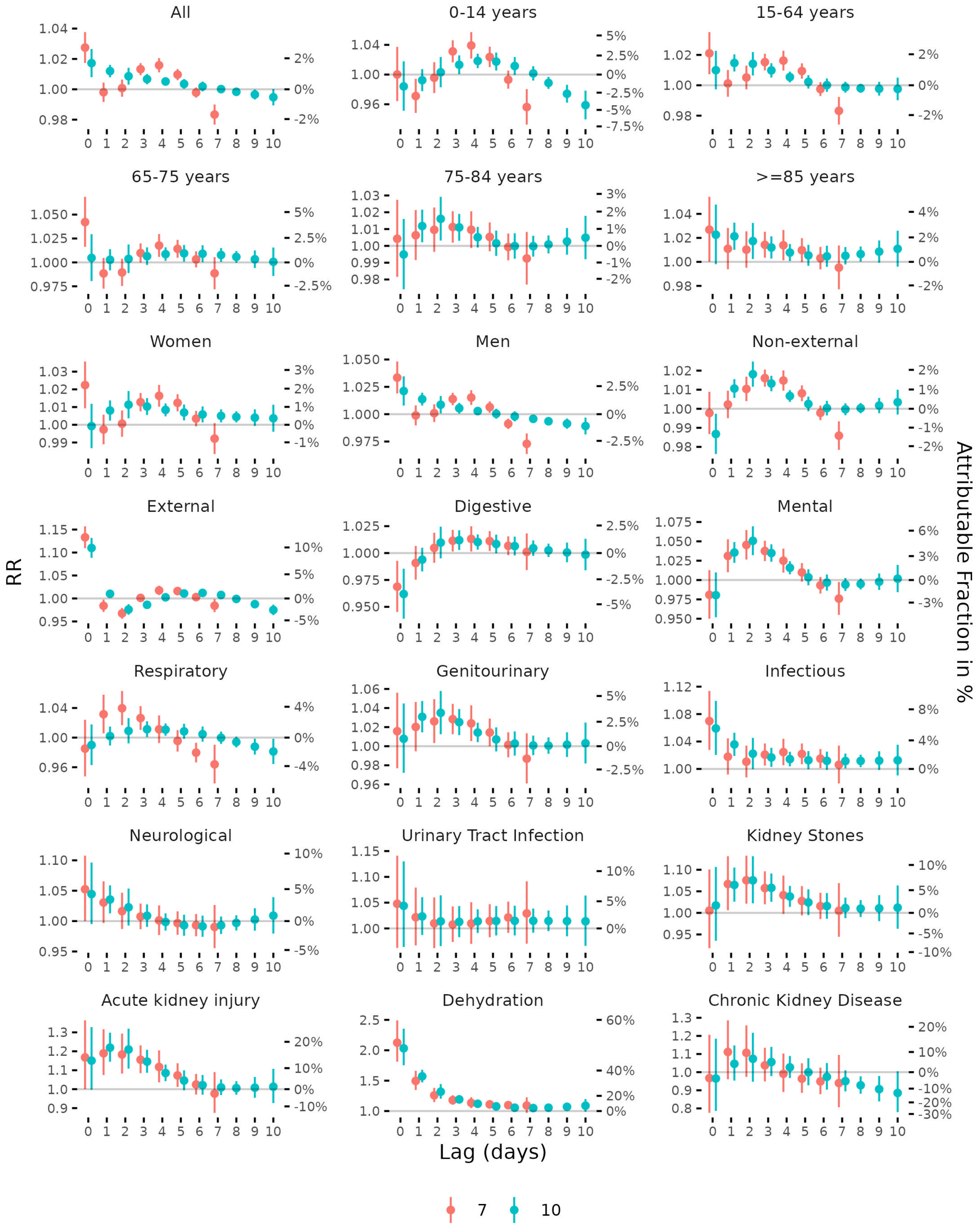

Figure A2: Exposure-response functions for emergency hospital admissions during the warm season (May-September) in Switzerland 1998-2019 by age and gender. Dotted line: reference temperature.

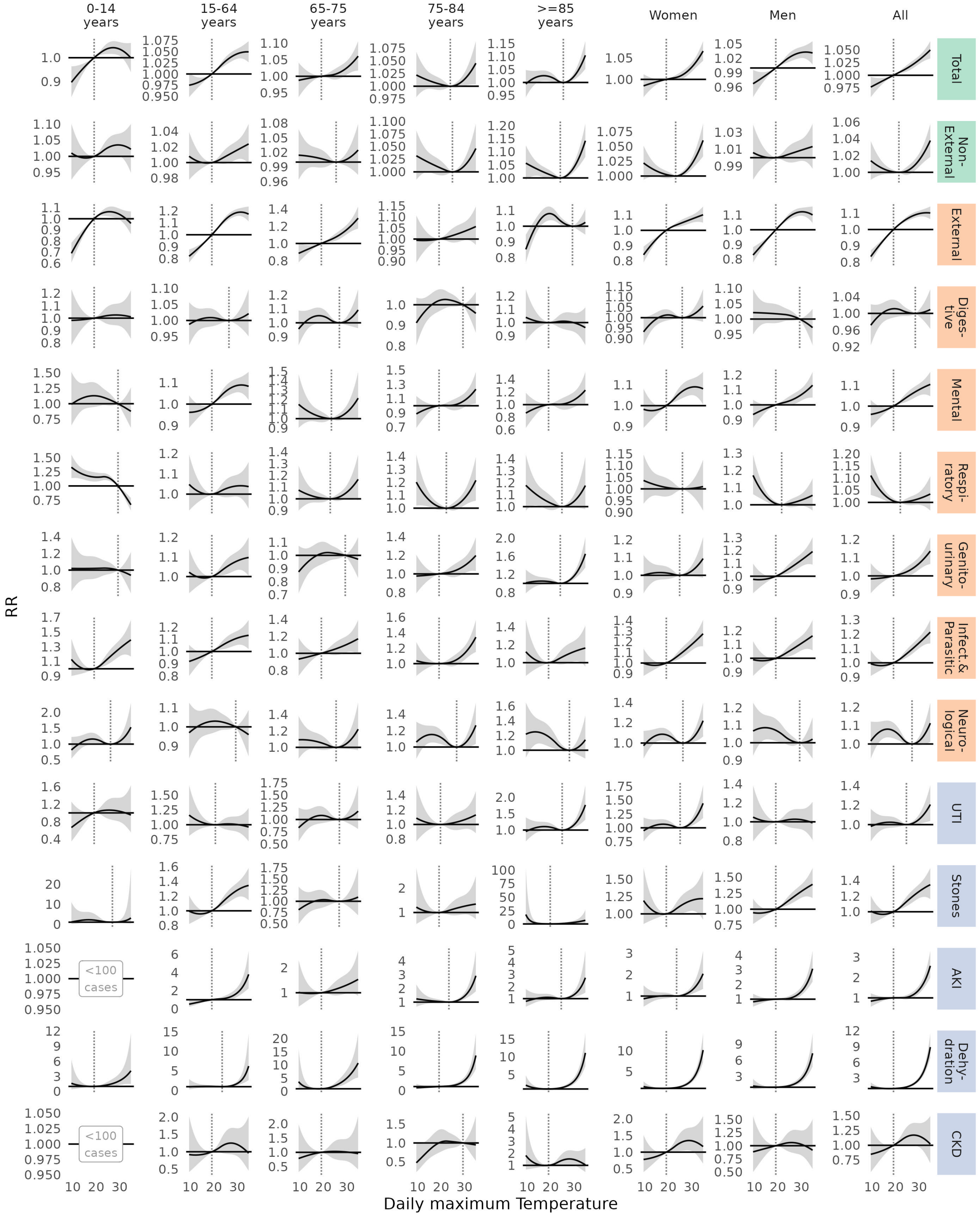

Figure A3: Sensitivity analysis: Exposure-Response-Functions, all age groups and diseases, for maximum, mean and minimum daily temperature. Knots: Percentiles of knots of the spline function. df : degrees of freedom for the temperature dimension. Dashed line: Reference temperature.

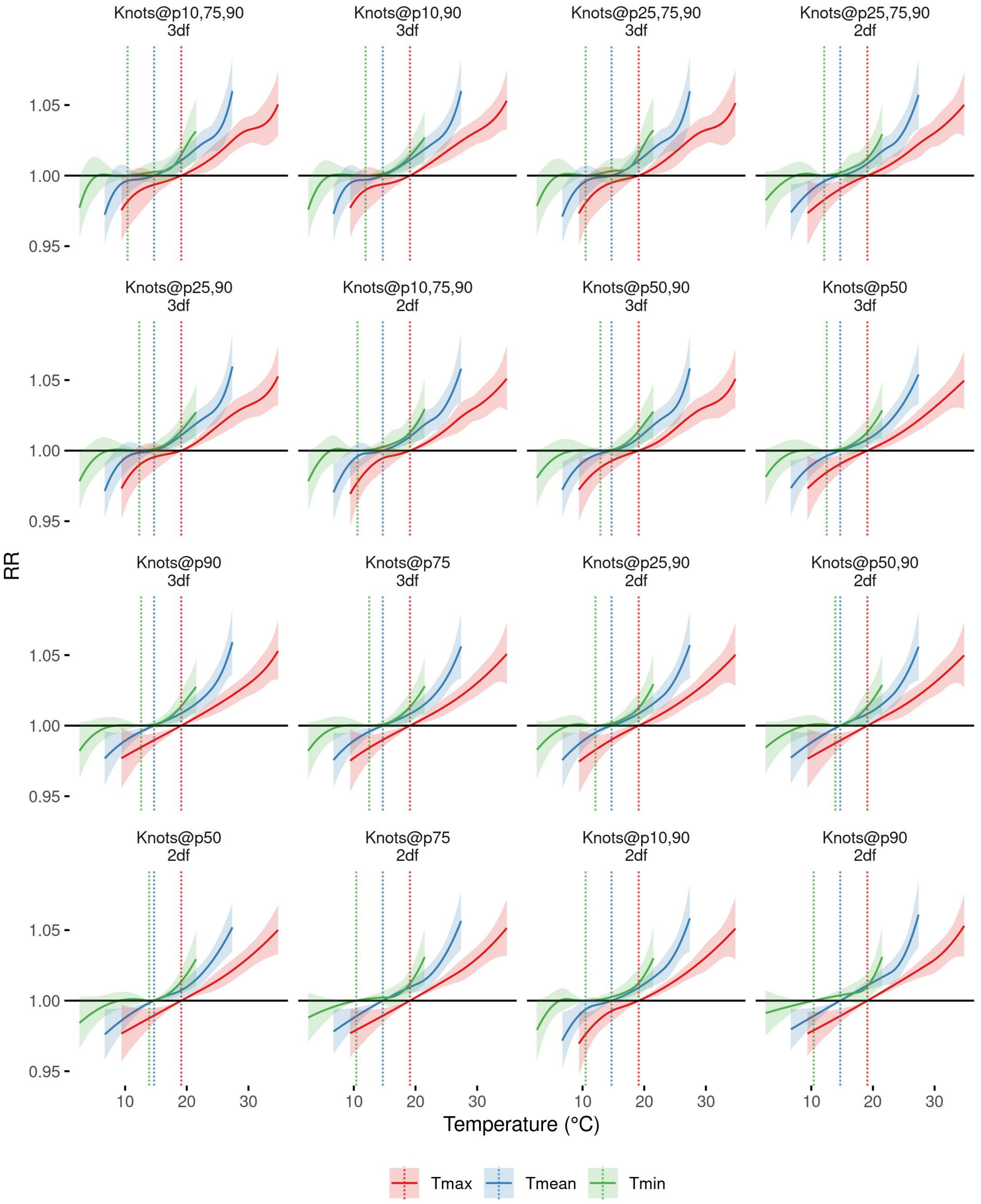

Figure A4: Sensitivity analysis - RRs comparing p99 (34°C) with reference temperature, main and alternative models. Alternative model parameters include different position of knots and degrees of freedom (df). Main model in blue (50_2, one knot at p50 + 2 degrees of freedom).

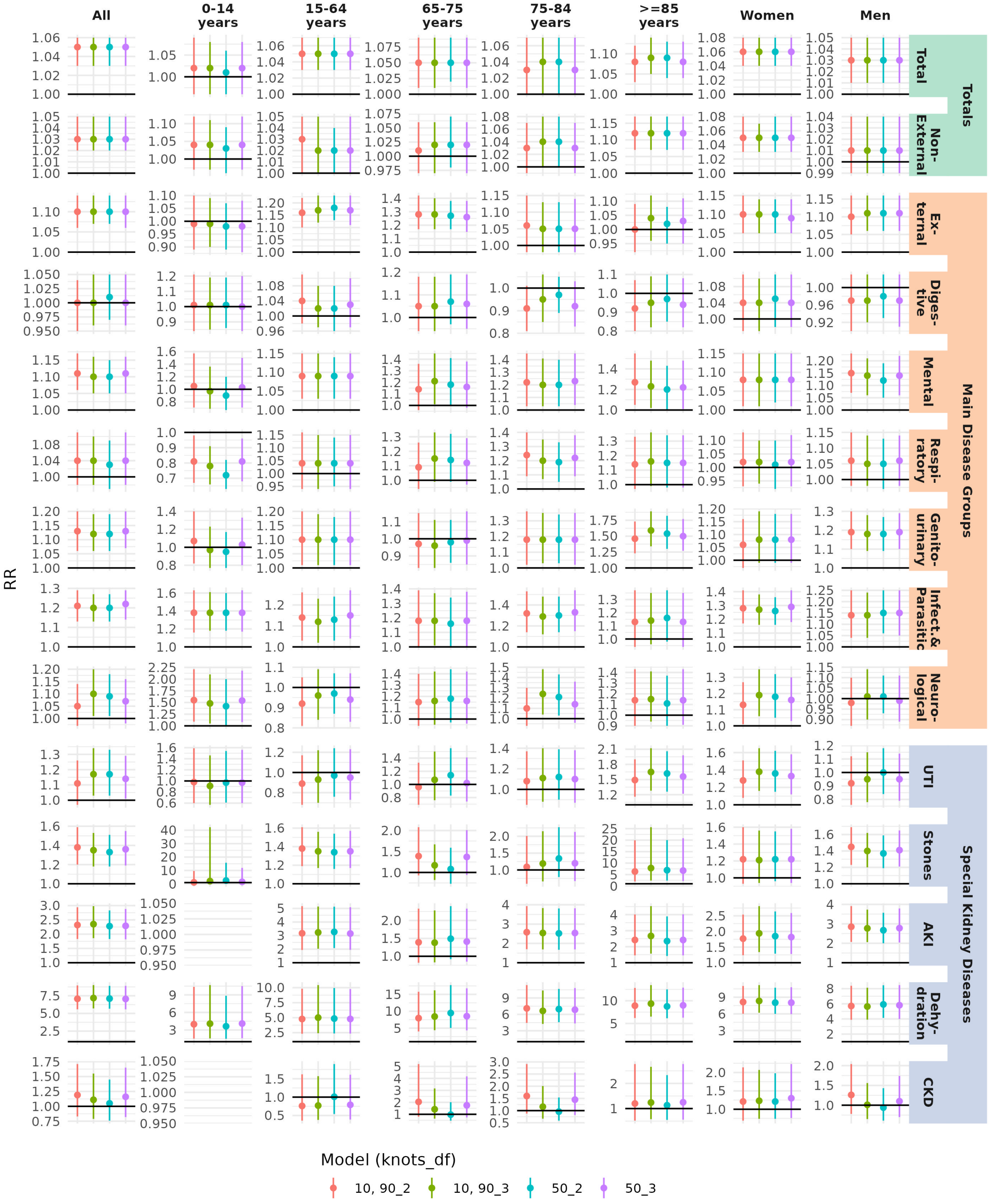

Figure A5: Sensitivity analysis - RRs for moderate (p90 / reference temperature) and extreme (p99 / p90) heat with main and alternative models. Alternative model parameters include different position of knots and degrees of freedom (df). Main model in blue (50_2: one knot at p50 & 2 degrees of freedom).

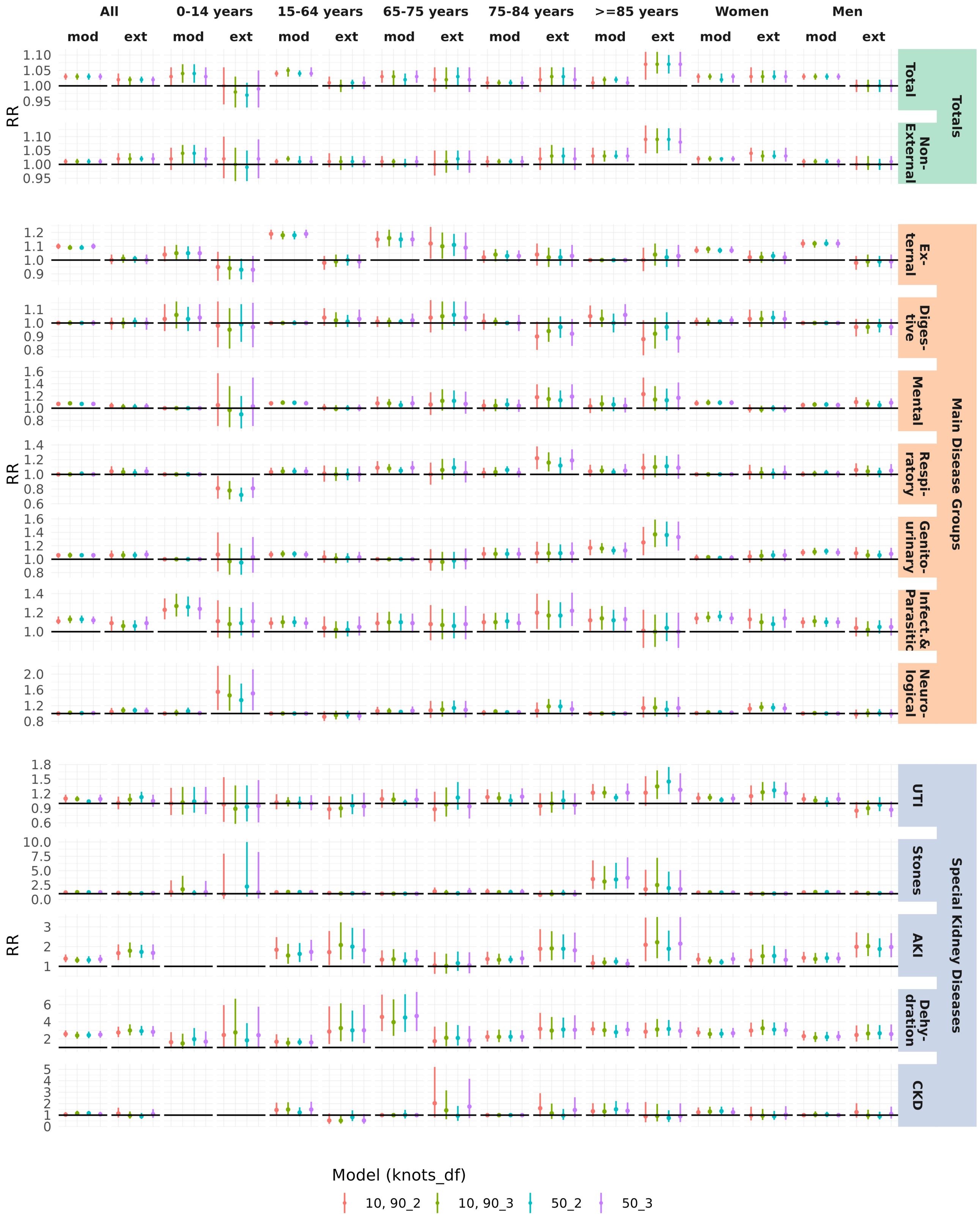

Table A1. Weather stations used in this study by canton, with median, minimum and maximum daily maximum temperature (Tmax)

Canton	Weather Station	median	min	max	Longitude/ Latitude	Elevation (m)
St. Gallen	St. Gallen	19.6	3.2	33.3	9°24/47°26	775
Zürich	Zürich / Fluntern	21.9	5.7	36.0	8°34/47°23	555
Bern	Bern / Zollikofen	22.2	5.9	37.0	7°28/46°59	552
Luzern	Luzern	22.5	6.8	35.8	8°18/47°02	454
Waadt	Pully	22.7	6.8	37.1	6°40/46°31	455
Basel-Land	Basel / Binningen	23.1	7.6	38.6	7°35/47°32	316
Basel-Stadt	Basel / Binningen	23.1	7.6	38.6	7°35/47°32	316
Genf	Genève / Cointrin	23.9	8.2	39.7	6°08/46°15	410
Tessin	Locarno / Monti	25.2	8.0	37.9	8°47/46°10	366
